# A surrogate gradient spiking baseline for speech command recognition

**DOI:** 10.3389/fnins.2022.865897

**Published:** 2022-08-22

**Authors:** Alexandre Bittar, Philip N. Garner

**Affiliations:** ^1^Idiap Research Institute, Martigny, Switzerland; ^2^École Polytechnique Fédérale de Lausanne, Lausanne, Switzerland

**Keywords:** spiking neurons, physiologically plausible models, deep learning, signal processing, speech recognition, surrogate gradient learning, artificial intelligence

## Abstract

Artificial neural networks (ANNs) are the basis of recent advances in artificial intelligence (AI); they typically use real valued neuron responses. By contrast, biological neurons are known to operate using spike trains. In principle, spiking neural networks (SNNs) may have a greater representational capability than ANNs, especially for time series such as speech; however their adoption has been held back by both a lack of stable training algorithms and a lack of compatible baselines. We begin with a fairly thorough review of literature around the conjunction of ANNs and SNNs. Focusing on surrogate gradient approaches, we proceed to define a simple but relevant evaluation based on recent speech command tasks. After evaluating a representative selection of architectures, we show that a combination of adaptation, recurrence and surrogate gradients can yield light spiking architectures that are not only able to compete with ANN solutions, but also retain a high degree of compatibility with them in modern deep learning frameworks. We conclude tangibly that SNNs are appropriate for future research in AI, in particular for speech processing applications, and more speculatively that they may also assist in inference about biological function.

## 1. Introduction

Recent years have seen the success of artificial neural networks (ANNs) in speech processing technologies. The neurons traditionally used in these modern networks take real numbers as inputs and produce real-valued outputs. In biological neurons, the information is transmitted in the form of binary sequences of events, called spike trains. The neurons in ANNs can be seen as an instantaneous firing rate approximation of biological spiking neurons, so that the information about the individual timings of the spikes is neglected. Several neuroscience studies suggest that precise spike timings are important in transmitting information, especially in the visual cortex and in auditory neurons (Mainen and Sejnowski, 1995; Van Rullen and Thorpe, 2001; Butts et al., 2007; Gollisch and Meister, 2008). With the idea of simulating brain-like networks to process information, this firing rate interpretation of spikes can be improved to spiking neuron models. Physiologically plausible mathematical models have been developed to describe the neuronal dynamics (Gerstner and Kistler, 2002; Izhikevich, 2007). The resulting spiking neurons constitute the building blocks of spiking neural networks (SNNs), and have been called the third generation of neural network models (Maass, 1997). The temporal dimension of spike trains make them naturally adapted to sequential input data, such as speech, for which, SNNs in principle may have a higher representation capability compared to traditional ANNs (Kasabov, 2019). In current practice however, only rarely do SNNs outperform ANNs (Leng et al., 2018; Moraitis et al., 2020). Another motivation toward SNNs is that the sparsity of spikes over time can allow energy-efficient hardware implementations (Davies et al., 2018; Roy et al., 2019; Dellaferrera et al., 2020; Panda et al., 2020), stimulated by event-based sensors, resulting in portable, low-powered devices. As pointed out by Pfeiffer and Pfeil (2018), this constitutes an advantage over conventional ANNs that rely on energy consuming high-end GPUs. Recent work by Jeffares et al. (2022) has shown that spike-based techniques can not only improve the ANN efficiency but also its task performance.

ANNs are most commonly trained using stochastic gradient descent (SGD), which relies on the chain rule of derivatives. During the forward pass, a batch of input examples is passed through the network, and a loss function is applied to the final outputs. During the subsequent backward pass, the network trainable parameters are updated to minimize the loss. The network gradually adapts to the task at hand by repeating this operation over a data set of prepared examples. SNNs are not directly compatible with gradient descent owing to their non-differentiable threshold behavior. Different methods have been developed to alleviate the problem. In particular, the surrogate gradient approach allows SNNs to be trained like recurrent ANNs using the Back-Propagation Through Time (BPTT) algorithm, which is a generalization of gradient descent to process sequential data. Recurrent neural networks (RNNs) have proven to be efficient on speech recognition tasks. In general, the reported performance of SNNs is inferior to that of the best ANNs (Wu et al., 2018, 2020; Cramer et al., 2020; Yin et al., 2020, 2021; Shaban et al., 2021; Yao et al., 2021), even if the gap is gradually closing. There is in fact theoretical (Maass, 1997; Moraitis et al., 2020; Perez-Nieves et al., 2021) and recent experimental (Moraitis et al., 2020) evidence that SNNs can outperform ANNs.

With this work, we aim to make SNNs available to a speech processing audience, the frameworks of which are currently dominated by ANNs. For this reason, we first include a quite thorough review-like introduction to the spiking neuron concepts that are compatible with these ANN frameworks. Subsequent experiments on speech command recognition are sufficient to define an appropriate framework and tools to demonstrate the utility of SNNs, whilst clearly being a prerequisite for more involved speech processing tasks. We also discuss potential energy advantages for integration into low-powered devices. Our first aim is therefore,

1. To identify which SNN training techniques are compatible with successful modern ANN frameworks, and establish a method that is able to compete with the ANN performance, whilst retaining the advantages of energy efficiency.

More generally, we aim

2. To assess the general capability of SNNs, and how they might represent an attractive alternative to standard ANNs.
3. To use a physiologically plausible approach to provide some insights on how the corresponding biological mechanisms in humans might be functioning.

Spiking versions of the Heidelberg Digits (SHD) and Google Speech Command (SSC) datasets have recently been released using physiological models of the cochlea (Cramer et al., 2020). We use these as well as their respective non-spiking, traditional versions (HD and SC) to conduct experiments with both SNNs and ANNs. Using a recovery current instead of the more conventional moving threshold formulation of Bellec et al. (2018), our implementation of adaptive neurons seems to convincingly improve upon previous efforts on similar tasks (Yin et al., 2020, 2021; Salaj et al., 2021; Shaban et al., 2021), as we achieve new state-of-the-art results with SNNs. Furthermore, a comparison with gated recurrent ANNs shows that our spiking baseline is capable of achieving competitive results, even without resorting to recurrent connections, showing the strength of a physiologically plausible approach.

We will start by introducing the mathematical models used to describe spiking neurons in Section 2.1. We will then show how ANNs are typically implemented (Section 2.2), to then build an equivalent forward pass for SNNs (Section 2.3). We will then focus on the training methods for SNNs in Section 2.4 and define our selected approach using surrogate gradients. To complete the description of our spiking networks, the loss function, and readout layer will be defined in Section 2.5. We will then explain the different speech perception tasks on which we conduct the experiments in Section 2.6, and finally present and discuss our results in Sections 3 and 4.

## 2. Materials and methods

### 2.1. Single spiking neuron models

The neuron models presented below will be used as building blocks of potentially deep spiking neural networks. In order to achieve a satisfying degree of compatibility with the modern frameworks developed for machine learning, our analysis will focus on single neuron models that rely on a limited number of parameters and are not excessively expensive in terms of computations.

#### 2.1.1. Leaky integrate and fire

The simplest and most widely used single neuron model is the leaky integrate and fire (LIF), the origin of which dates back to the beginning of the twentieth century with the work of Lapicque (1907). The dynamics of a single neuron are described by the membrane potential *u*(*t*), which evolves in time as a function of some input current *I*(*t*). In the absence of stimuli, i.e., when *I*(*t*) = 0, the membrane potential *u*(*t*) decays exponentially to some resting value *u*_rest_ with a time constant τ_*u*_ ≈ 10ms. When *I*(*t*) ≠ 0, the membrane potential *u*(*t*) integrates the incoming stimuli and increases or decreases accordingly. As presented by Gerstner and Kistler (2002), the dynamics in continuous time follow the differential equation,


(1) $$\tau_{u}\dot{u}(t)=-(u(t)-u_{\text{rest}})+RI(t),$$

where *R* is the membrane resistance. In order to have spikes, a threshold value ϑ must be added to the model, so that when the potential reaches the critical value, a spike is emitted and the potential is reset to a new value *u*_*r*_ < ϑ.


(2) $$\begin{array}{l} \text{if }u(t=t^{f})\geq \vartheta \text{ then }s(t^{f})=1\\ \text{ and }\underset{\delta \to 0;\delta >0}{\lim}u(t^{f}+\delta )=u_{r}. \end{array}$$

#### 2.1.2. Adding an adaptation variable

Although widely used, the LIF model is not sufficient to reproduce many of the various firing patterns observed in biological neurons, such as adaptive, bursting, transient, and delayed (Gerstner and Kistler, 2002). The idea of a second equation to describe an adaptation (or accommodation) variable between threshold and subthreshold voltage can be traced back to Hill (1936). The work of Treves (1993), Izhikevich (2001), and Brunel et al. (2003) have notably lead to its modern formulation, in which a recovery variable *w*(*t*) is linearly coupled to the membrane potential *u*(*t*) in the subthreshold regime, and a mechanism is used for spike-triggered adaptation. The resulting more complex neuronal dynamics of an adaptive, linear LIF model (adLIF) follow the differential equations,


(3) $$\begin{array}{l} \tau_{u}\dot{u}(t)=-(u(t)-u_{\text{rest}})-Rw(t)+RI(t)\\ -\tau_{u}(\vartheta -u_{r})\sum_{f}\delta (t-t^{f}) \end{array}$$

(4) $$\tau_{w}\dot{w}(t)=-w(t)+a(u(t)-u_{\text{rest}})+\tau_{w}b\sum_{f}\delta (t-t^{f}),$$

where the adaptation current typically evolves more slowly than the potential, i.e., with a longer time constant τ_*w*_ ≈ 100 ms compared to τ_*u*_ ≈ 10 ms. When the potential reaches a peak value *u*(*t*) = ϑ, its displacement is considered large enough to represent a spike, which defines the firing time *t*^*f*^ = *t*. The potential is then reset, $u\left(t^{f}\right)=u_{r}$ and the recovery variable pushed by an amount *b*, *w*(*t*^*f*^) = *w*(*t*^*f*^)+*b*, which was here directly included in the differential equations using delta-functions. The resulting adaptive neurons exhibit subthreshold adaptation characterized by the parameters *a* and τ_*w*_, and spike-triggered adaptation, regulated by the jump size *b*. It is worth noting that this form of adaptation does not include a moving or dynamic threshold (Fuortes and Mantegazzini, 1962; Chacron et al., 2003; Badel et al., 2006; Jolivet et al., 2006).

#### 2.1.3. Adding a nonlinearity

Using a nonlinearity instead of the linear relation to the membrane potential in Equation (3) transforms the strict voltage threshold into a more biologically plausible smooth spike initiation zone (Brette and Gerstner, 2005). For such adaptive, nonlinear LIF models, the complete dynamics can be represented by a trajectory *u*(*t*), *w*(*t*) on a 2D-plane. Before receiving any stimuli, the neuron is at equilibrium on a stable fixed point. Depending on the model's parameters (*a*, *b*, *u*_*r*_, *u*_rest_, *R*, τ_*u*_, and τ_*w*_), a particular set of incoming spike trains can cause the trajectory to go through a *bifurcation* and form a limit cycle, resulting in repetitive firing. The neuron model of Izhikevich (2003) uses a quadratic function,


(5) $$\begin{array}{r} f\left(u\right)=-u\left(t\right)-u_{\text{rest}}\vartheta -u\left(t\right), \end{array}$$

and the adaptive exponential integrate and fire model (AdEx) of Brette and Gerstner (2005) uses a combination of linear and exponential functions,


(6) $$f(u)=-(u(t)-u_{\text{rest}})+\Delta \exp[-\frac{\vartheta -u(t)}{\Delta }].$$

This last neuron model seems to be the most physiologically plausible in terms of fitting with naturalistic pyramidal-neuron voltage traces (Badel et al., 2008).

#### 2.1.4. Discrete time formulation

Let us come back to the linear adLIF model presented in 2.1.2. By assuming *u*_rest_ = *u*_*r*_ and making the changes of variables $u\to \frac{u-u_{\text{rest}}}{\vartheta -u_{\text{rest}}}$, $w\to \frac{Rw}{\vartheta -u_{\text{rest}}}$ and $I\to \frac{RI}{\vartheta -u_{\text{rest}}}$, Equations (3) and (4) can be rewritten in discrete time, using a forward-Euler first-order exponential integrator method with a step size of Δ*t* = 1ms, resulting in the following forward pass in which all quantities are dimensionless,


(7) $$\begin{array}{rc} u\left[t\right] & =\alpha u\left[t-1\right]+\left(1-\alpha \right)I\left[t\right]-w\left[t-1\right]-\vartheta s\left[t-1\right] \end{array}$$

(8) $$\begin{array}{rc} w\left[t\right] & =\beta w\left[t-1\right]+\left(1-\beta \right)au\left[t-1\right]+bs\left[t-1\right] \end{array}$$

(9) $$\begin{array}{rc} s\left[t\right] & =u\left[t\right]\geq \vartheta . \end{array}$$

Here the neuron parameters have been redefined as *a*→*Ra*∈[−1, 1], $b\to \frac{Rb}{\vartheta -u_{\text{rest}}}\in \left[0,2\right]$, α: = exp(−Δ*t*/τ_*u*_)∈[0.60, 0.96], β: = exp(−Δ*t*/τ_*w*_)∈[0.96, 0.99], $\vartheta \to \frac{\vartheta -u_{\text{rest}}}{\vartheta -u_{\text{rest}}}=1$ and $u_{r}\to \frac{u_{r}-u_{\text{rest}}}{\vartheta -u_{\text{rest}}}=0$ based on physiologically plausible ranges of values. In Equations (7) and (8), the first term describes the leak, the second the excitation, and the third the effect of having spiked at the previous time step. Equation (9) produces a 0 or a 1 when the membrane potential is below or above threshold. The LIF model can then be viewed as a simplification of the adLIF with *a* = *b* = 0, so that there is no recovery current *w*[*t*] = 0 = constant, and the only tunable neuron parameter is α.

### 2.2. Artificial neural networks

The vast majority of neural networks used in modern machine learning tasks are organized in layers of artificial neurons from the second generation as defined by Maass (1997). Here we are interested in networks that can process sequential inputs, in particular speech. Starting from some discrete signals $y_{j}^{0}\in {\mathbb{R}}^{T}$ of length *T* and time step Δ*t*, *j* = 1, …, *N*^0^, a standard ANN processes the information layer by layer, as follows. In the *l*-th layer, a single non-spiking artificial neuron *i* receives inputs from neurons *j* = 1, …, *N*^*l*−1^ of the previous layer. If recurrent connections are enabled, neuron *i* also receives inputs from all neurons *k* = 1, …, *N*^*l*^ in the same *l*-th layer. The overall stimulus of neuron *i* in layer *l* at time step *t* is then computed as,


(10) $$\begin{array}{c} I_{i}^{l}[t]=\sum_{j=1}^{N^{l-1}}W_{ji}^{l}y_{j}^{l-1}[t]+\sum_{k=1}^{N^{l}}V_{ki}^{l}y_{k}^{l}[t-1]+b_{i}^{l}, \end{array}$$

where *W*^*l*^ and *V*^*l*^ are the trainable feedforward and recurrent weight matrices respectively, and *b*^*l*^ is a trainable bias vector. The network is called a multilayer perceptron (MLP) if only feedforward connections are implemented, i.e., *V* = 0, and a recurrent neural network (RNN) when additional recurrent connections are present, i.e., *V*≠0. In both cases, the sequential output of the neuron $y_{i}^{l}\in {\mathbb{R}}^{T}$ is simply computed using a nonlinear activation function *g*(·),


(11) $$\begin{array}{r} y_{i}^{l}\left[t\right]=g\left(I_{i}^{l}\left[t\right]\right), \end{array}$$

which produces real-valued signals. Using a sigmoid activation function *g*(*x*) = (1 + *e*^−*x*^)^−1^ for instance, the analog neuron output *y* ∈ [0, 1] can be interpreted as the firing rate of a spiking neuron (over some arbitrary period of time).

The most successful recurrent architectures are based on the long short-term memory (LSTM), defined by Hochreither and Schmidhuber (1997), in which gates are used to filter out irrelevant information and tackle the vanishing/exploding gradient problem. Each gate uses its own distinctive feedforward and recurrent weights, which increases the total number of trainable parameters. The gated recurrent unit (GRU) of Cho et al. (2014) and the light GRU (liGRU) of Ravanelli et al. (2018) constitute gradual simplifications of the LSTM with fewer gates in an effort to reduce the size of recurrent units. Very recently, the authors have derived a probabilistically interpretable version of the liGRU called light Bayesian recurrent unit (liBRU) that showed slight improvements over the liGRU on speech recognition tasks (Bittar and Garner, 2021). We will implement MLPs, RNNs, liBRUs, and GRUs, which will serve as an ANN-baseline to compare with our SNNs.

### 2.3. Spiking neural networks

In a spiking neural network, a single neuron *i* in the *l*-th layer receives pre-synaptic inputs from neurons *j* = 1, …, *N*^*l*−1^ of the previous layer in the form of spike trains $s_{j}^{l-1}\in {\left\{0,1\right\}}^{T}$. If recurrent connections are enabled, it also receives spike trains $s_{k}^{l}$ from all other neurons *k* = 1, …, *N*^*l*^, *k*≠*i* in the same *l*-th layer. The overall stimulus of neuron *i* in layer *l* can then be written as


(12) $$\begin{array}{c} I_{i}^{l}[t]=\sum_{j=1}^{N^{l-1}}W_{ji}^{l}s_{j}^{l-1}[t]+\sum_{k=1;k\neq i}^{N^{l}}V_{ki}^{l}s_{k}^{l}[t-1]+b_{i}^{l}, \end{array}$$

where the weight matrices *W*^*l*^ and *V*^*l*^ correspond to the strength of the synaptic connections, and the bias *b*^*l*^ to heterogeneous resting values of the membrane potential among neurons. The excitatory and inhibitory connections between physiological neurons are here represented by positive and negative weights, respectively. After firing, a biological neuron enters a period of refractoriness so that it cannot immediately fire a second spike. In order to maintain this physiological feature of self inhibition, the diagonal elements of *V* can be set to 0 in Equation (12), as positive diagonal weights would act against refractoriness. The latter can be then modeled *via* the reset of the membrane potential to a value *u*_*r*_ after spiking. Alternatively, negative diagonal elements could be used to directly decrease the value of the membrane potential. With Equations (10) and (12), we see that the stimulus of a spiking neuron can be computed in the exact same way as for a standard artificial neuron.

The forward pass through an SNN is then defined by vectorizing Equations (7)–(9) and looping them over layers and time. The main difference with ANNs is therefore that the dynamics of the membrane potential, combined with the threshold behavior replace the simple activation function of Equation (11) and produce binary signals *s*^*l*^∈{0, 1}^*T*×*Nl*^ instead of analog ones *y*^*l*^∈ℝ^*T*×*Nl*^. As pointed out by Neftci et al. (2019), such dynamics can be viewed as a nonlinear activation function which makes SNNs a special case of RNNs. In this paper, however, the term RNN describes a purely non-spiking recurrent network, as defined in Section 2.2.

In reaction to a given stimulus *I*[*t*], different sets of values for the neuron parameters α, β, *a* and *b* will lead to different firing patterns, which is an important particularity of SNNs. In ANNs, since the same activation function is typically applied to all neurons, as written in Equation (11), two neurons receiving the same stimulus will always produce the same output. As demonstrated by Perez-Nieves et al. (2021), the introduction of heterogeneity in the spiking neuron parameters can considerably improve the network performance, especially for tasks that have a rich temporal structure. This form of neural heterogeneity therefore seems to represent a theoretical advantage of SNNs over standard ANNs on such tasks, as it may allow superior representations of the temporal information. As reviewed by Apicella et al. (2021), trainable or adaptable activation functions have also been used inside ANNs, and are known to improve their accuracy. In this paper however, we do not attempt to additionally cover this large field. The above review points out that the enabled improvements can usually be replicated using a more conventional non-trainable homogeneous activation function, and simply more neurons or layers. Also with the idea of being representative of the currently established standard practice in the field, our ANNs use a homogeneous activation function as defined in Equation (11). As our own ANN implementations sometimes outperform those of the literature (De Andrade et al., 2018; Cramer et al., 2020), we believe that they form an adequate ANN baseline for comparing with SNNs.

With the objective of assessing the capabilities of spiking neurons compared to standard artificial ones, we must define equivalent architectures, that only differ in the type of neurons that they employ. As we will see in 3.5, even though the spiking neuron parameters α, β, *a*, and *b* can be made trainable, the total number of trainable parameters in a network remains largely dominated by the amount of connecting weights. This means that in terms of the total number of trainable parameters, SNNs with and without recurrent connections are comparable to RNNs and MLPs respectively. On the other hand, state-of-the-art gated RNNs remain considerably larger and do not have any direct spiking equivalent in this study. It is also worth mentioning that even though purely feedforward SNNs do not have recurrent connections, they still include a form of unit-wise recurrence from Equations (7) and (8), where a dependence to the previous time step is present in the dynamics of the membrane potential. This implies that non-recurrent SNNs are theoretically capable of developing a form of memory, without the need of recurrent connections, which is not the case for MLPs.

### 2.4. Training methods for SNNs

Biological neurons exhibit activity-dependent synaptic plasticity characterized by long term potentiation and depression. This behavior can be modeled using a form of spike timing dependent plasticity (STDP), as defined by Dan and Poo (2006). Without the need of labeled examples, this form of unsupervised Hebbian learning is sufficient to detect correlations in the input stimuli and learn encodings of real-world data. However, in order to perform motor tasks, STDP must be combined with a form of global reward-based learning that involves neuromodulators in the brain (Schultz et al., 1997; Schultz, 2007, 2010; Frémaux and Gerstner, 2016).

Artificial neurons, on the other hand, are most commonly trained using stochastic gradient descent (SGD). This technique compares the model predictions to desired outputs on a batch of examples *via* a loss function. The error of the whole batch is then back-propagated through the network using the chain-rule of derivatives, and the trainable parameters of the entire network are updated accordingly. This form of supervised, global and offline learning is however highly biologically implausible (O'Reilly and Munakata, 2000). In comparison, the weight adjustment with STDP happens *online*, i.e., each time a spike is emitted, and has only a local dependence on the pre- and post-synaptic neurons. Nevertheless, SGD represents the most successful training algorithm used in ANNs. With the aim of evaluating the compatibility of SNNs within ANN frameworks, we will focus on training SNNs with gradient descent.

Using SGD with SNNs is challenging because the derivative of the spike function in Equation (9) with respect to the membrane potential is zero in the subthreshold regime (when no spikes are emitted, i.e., almost everywhere) and undefined when threshold is reached and a spike is produced. Moreover, small perturbations of the synaptic weights can either lead to considerably different output spike trains, or produce no change at all. The numerous discontinuities caused by the threshold mechanism make the search of a global optimum particularly difficult. This problem is especially visible when training multi-layered architectures with SGD. Nevertheless, training SNNs with SGD can still be achieved through a variety of approaches that can be grouped into the following three general categories:

1. Using a hybrid ANN-SNN model
2. Using a differentiable model
3. Using surrogate gradients.

#### 2.4.1. Hybrid ANN-SNN models

The first category, which has been reviewed by Abbott et al. (2016), circumvents the problem of training SNNs by using conventional rate-based ANNs instead. The resulting architectures do exhibit a spiking behavior during the forward pass, but the spike timings are ignored in the learning rule. Recently, Wu et al. (2021) have managed to ensure an efficient gradient based back-propagation by coupling an SNN with an ANN through layer-wise weight sharing. During the forward pass, the SNN computes the exact spiking neural representations, and the ANN the corresponding approximate spike counts (or firing rates). During the backward pass, the error is backpropagated through the ANN *via* SGD and the weight updates are transferred to the SNN. This tandem learning technique allows fast and efficient learning with multi-layered architectures and has notably proven to be successful on speech recognition tasks (Wu et al., 2020). Nevertheless, one could argue that the information about the timings of the individual spikes is still reduced to a rate-based approximation during the backward pass. Moreover, this approach so far neither includes adaptive neuron models nor recurrent connections.

#### 2.4.2. Differentiable neuron models

The second category, which has been reviewed by Neftci et al. (2019), involves soft-threshold models (Hodgkin and Huxley, 1952; FitzHugh, 1961; Morris and Lecar, 1981; Huh and Sejnowski, 2018), probabilistic models (Jang et al., 2019), spike train convolution models (Lin et al., 2017; Lin and Shi, 2018; Wang et al., 2019) as well as single-spike timing-based models (Bodyanskiy and Dolotov, 2013; Mostafa, 2017; Comsa et al., 2020). These neuron models are interesting, but beyond the scope of this paper, that focuses on the non-differentiable models presented in 2.1.

#### 2.4.3. Surrogate gradient methods

Also presented by Neftci et al. (2019), the problem of the non-differentiable threshold behavior can be solved using surrogate gradients. During the backward pass, the Heaviside step function of the spike generation is smoothed into a suitable differentiable function. With this approach, the threshold operation is only approximated during the backward pass, and remains a step function inside the forward computations. The derivative has notably been approximated using a rectifying linear unit (Bohte et al., 2002), a sigmoid derivative (Schrauwen and Van Campenhout, 2006; Zenke and Ganguli, 2018), an exponential function (Shrestha and Orchard, 2018), a piecewise linear function (Bellec et al., 2018; Panda et al., 2020), a Gaussian (Yin et al., 2020), a multi-Gaussian (Yin et al., 2021) and a boxcar function (Kaiser et al., 2020). An SNN can then be considered as a special case of a recurrent neural network (RNN) and the error Back-Propagation Through Time (BPTT) algorithm becomes applicable. Nevertheless, the sparsity in time of non-zero gradients, combined with the problems of exploding/disappearing gradients remain. This third and last category of SGD-based training methods is rather versatile compared to the first two (2.4.1 and 2.4.2), as it is not limited to a specific neuron model and allows the use of the different spiking neuron models described in 2.1. We will therefore concentrate our analysis on the surrogate gradient approach, but still include a comparison with the tandem method of Wu et al. (2021) presented in 2.4.1.

**Figure 1:**
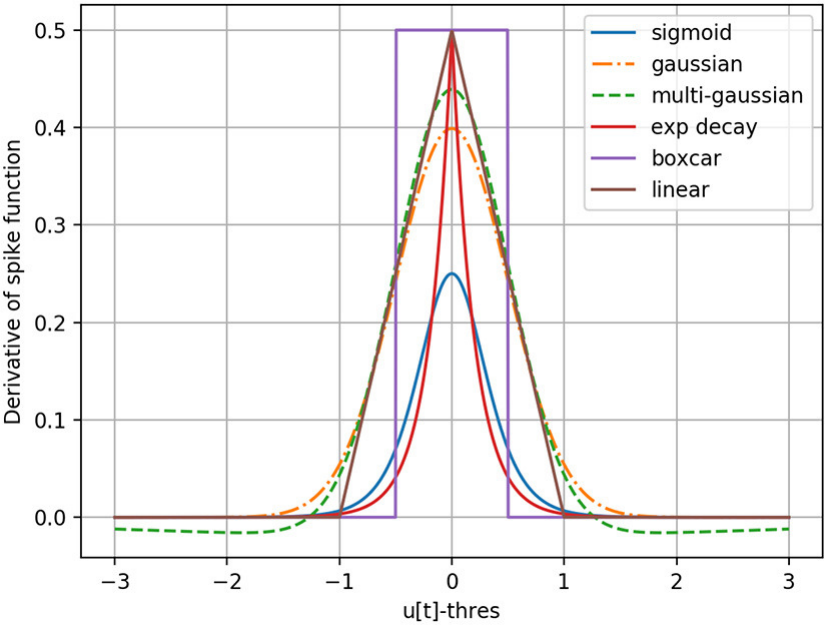
Different surrogate gradient functions to approximate the derivative of the step-function responsible for spike generation.

In an SNN as defined in Section 2.3, by exploiting auto-differentiation inside the deep learning framework PyTorch (Paszke et al., 2017), one can manually replace the undefined gradient of the step function in Equation (9) with a surrogate, and make the backward pass and therefore gradient descent possible for the whole network. Different choices of surrogate gradients illustrated in Figure 1 can be considered. For the purpose of this paper, the boxcar method, previously used by Kaiser et al. (2020), is chosen by default based on *ad-hoc* experiments. It is defined as,


(13) $$\frac{\partial s[t]}{\partial u[t]}=\{\begin{array}{ll} 0.5\; & \text{if }|u[t]-\vartheta |\;\leq 0.5\\ 0\; & \text{otherwise} \end{array}$$

and is quite inexpensive in terms of computations.

### 2.5. Loss function and readout layer

In order to use an SNN as a classifier that can be trained inside a typical ANN framework, we choose a cross-entropy loss function to be applied to the outputs of the final layer *L* of the architecture. Instead of a sequence of spikes, this readout layer must output one value *o*_*i*_ per neuron *i* = 1, …, *N*^*L*^ that indicates its level of activity over time. During inference, the neuron with the highest activity will be chosen. We considered four different methods for the readout layer,

1. a spiking layer using the spike count, $o_{i}=\overset{T}{\underset{t=1}{\sum }}s_{i}^{L}\left[t\right]$
2. a non-spiking layer using the last potential value over time, $o_{i}=u_{i}^{L}\left[T\right]$
3. a non-spiking layer using the maximal potential value over time, $o_{i}=\underset{t=1,\ldots ,T}{\max}u_{i}^{L}\left[t\right]$
4. a non-spiking layer using a cumulative sum of the potential over time, $o_{i}=\overset{T}{\underset{t=1}{\sum }}\text{softmax}\left(u_{i}^{L}\left[t\right]\right)$ .

In *ad-hoc* experiments (see Table 1), the last technique gave the best performance and is what is used in all presented results. Non-spiking LIF neurons with no recurrent connections are used inside the readout layers of all presented models.

**Table 1:** Results on the SHD dataset for SNNs with different types of readout layer.

**Network**	**Cumulative sum (%)**	**Spike count (%)**	**Last potential (%)**	**Max potential (%)**
LIF 3 × 128	87.27	**87.45**	62.45	77.99
LIF 3 × 512	**89.94**	87.27	67.74	79.46
adLIF 3 × 128	**93.06**	90.35	90.07	88.56
adLIF 3 × 512	**93.93**	93.52	90.53	89.61

Bold values indicate the best performing type of readout layer.

For the non-spiking baseline, the readout layer of RNNs was first defined as a recurrent layer, and the same cumulative sum used for SNNs was applied to its output sequence. Even though this might appear as the best choice for comparison with the SNN technique, we found that applying the cumulative sum in the penultimate (*L*−1) layer instead, followed by a final linear layer gave better results, which is what is used for all reported RNN, liBRU, and GRU results in the manuscript. For non-recurrent ANNs, the cumulative sum is simply applied to the final output sequences.

All models take inputs of size (*N, T, F*) and return outputs of size (*N, C*), where *N* is the batch size (i.e., the number of examples in one batch), *T* the number of time steps, *F* the number of input features/channels and *C* the number of classes (labels). The ground truths are given as a vector *y* of size (*N*) containing the label indexes. The cross-entropy loss is then computed as,


(14) $$ℒ=-\frac{1}{N}\sum_{n=1}^{N}\text{log}\frac{\text{exp}(o[n,y[n]])}{\sum_{c=1}^{C}\text{exp}(o[n,c])}.$$

The Adam optimizer (Kingma and Ba, 2015) is used for all experiments with initial learning rates of 0.01 and 0.001 on the spiking and non-spiking datasets, respectively. A scheduler is also defined to reduce the learning rate by a factor 0.7 if there is no improvement on the validation set accuracy during 10 epochs in a row. This approach proved to be suitable for both SNNs and ANNs and is employed in all presented networks.

**Figure 2:**
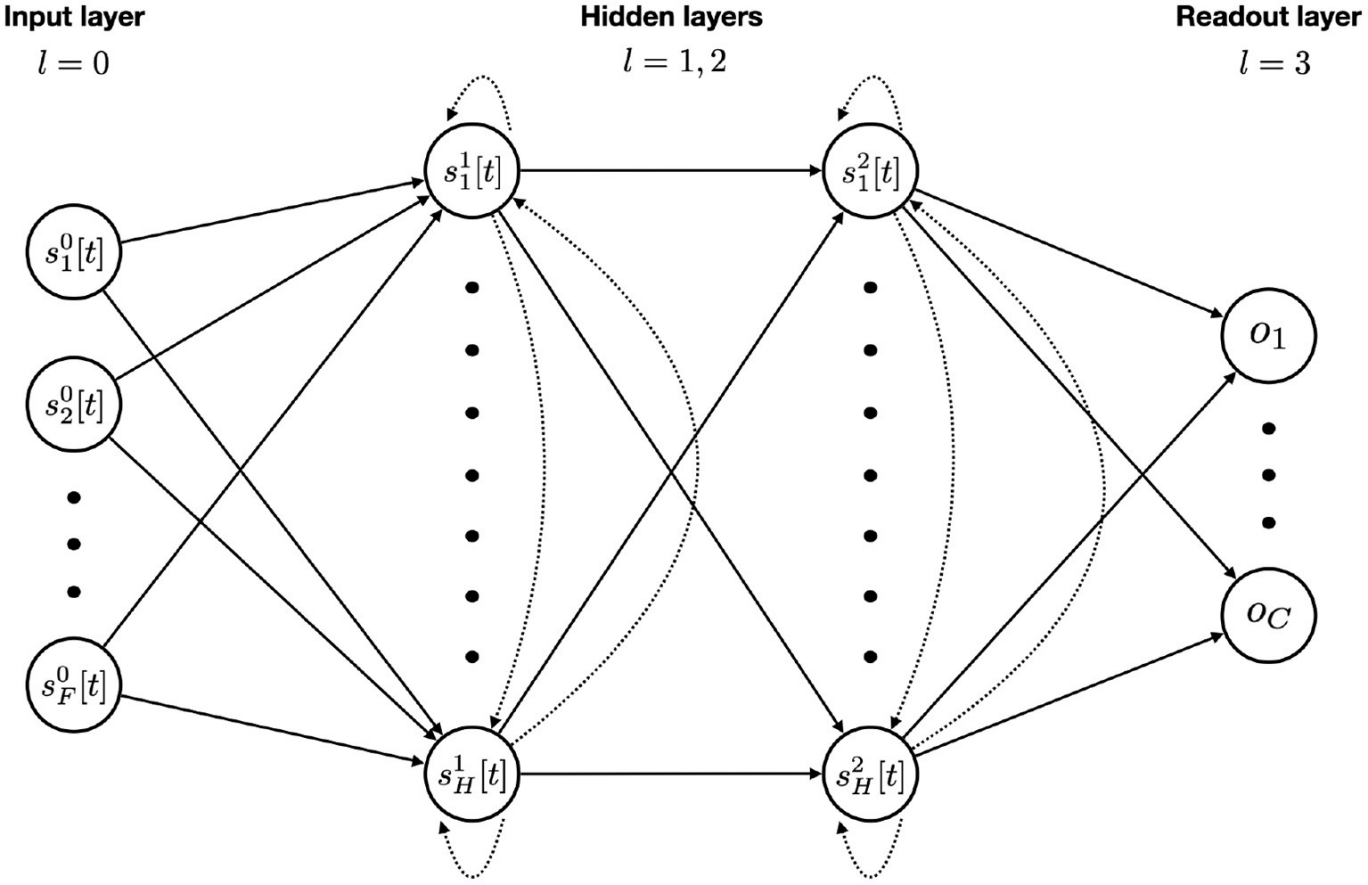
SNN model architecture with *F* input features, two recurrent layers with *H* hidden units each, and a final readout layer for the *C* classes. Feedforward and recurrent connections are represented with solid and dotted arrows respectively. An input unit is simply the value of that feature at the given time step. Each hidden unit integrates the incoming stimuli from feedforward and recurrent connections at time step *t* with Equation (12). The membrane potential is updated and a spike is emitted if threshold is reached. A readout unit integrates the purely feedforward incoming stimuli, updates its membrane potential (without spiking) and accumulates it over time using the softmax function. After passing the whole sequence through the network, the outputs then go into a cross-entropy loss function. Back-Propagation is made possible *via* the use of a boxcar surrogate gradient.

The general network architecture, used for all SNNs in this work, is presented in Figure 2. We will now explain the tasks on which they will be evaluated.

### 2.6. Speech perception tasks for SNNs

This research focuses on the bio-inspired processing of auditory information, leading to the formation of appropriate representations and the extraction of relevant features that can then be used for different tasks. The long-term objective is to perform automatic speech recognition (ASR) using a physiologically plausible approach that includes waveform to spike conversion followed by processing of the information *via* spiking neural networks.

However, ASR is a complex task; modern approaches involve end-to-end deep networks, whereas previous techniques needed to solve a series of subtasks, typically feature extraction, phoneme recognition and decoding. In the field of SNNs, it appears that one could benefit from first focusing on the simpler task of speech command recognition to better understand spiking networks. Whilst retaining the processing of auditory information, this more elementary task neither involves too many components in the pipeline, nor requires very deep networks and therefore constitutes a first necessary step in the direction of efficient ASR with SNNs.

We will start by giving a short summary of the biological processes involved in speech perception. We then review LAUSCHER, a bio-inspired model to convert audio waveforms into spike trains, and some resulting, newly available spiking datasets.

#### 2.6.1. From waveform to spikes

A speech utterance arrives at the ear in the form of air vibrations. From the eardrum it travels *via* the ossicles to the cochlea, hence the basilar membrane and the organ of Corti, ultimately stimulating hair cells that convert the physical movement into electrical signals. The signals take the form of spike trains on the auditory nerve. Many conventional ASR “filterbank” front-ends are rough analogs of this process, notably modeling the logarithmic response to frequency and to amplitude.

Cramer et al. (2020) have developed LAUSCHER, a biologically plausible cochlear model to convert audio waveforms into spike trains. A cochlear model, based on the models developed by Sieroka et al. (2006) is used to calculate the hydrodynamic shallow water basilar membrane response to the input waveform. The output of the cochlea then goes into a transmitter pool-based hair cell model, derived from the work of Meddis (1986, 1988). Finally, a layer of auditory neurons called bushy cells convert the signal to spike trains using LIF dynamics.

Such a framework allows a direct conversion from audio waveforms into spike trains, whilst solely relying on physiological processes. In order to train SNNs on speech data, the general and most commonly used alternative is to extract acoustic features from the waveform and interpret them as firing rates to produce spike trains *via* Poisson processes. Even though the latter approach still shows some physiological plausibility, a single firing rate value is used to produce spikes during the length of a frame (typically 25ms). This concession comes from the need of using datasets that were originally designed for ANNs, i.e., rate-based approximations of SNNs.

#### 2.6.2. Spiking datasets

In order to rectify the absence of free spike-based benchmark datasets, Cramer et al. (2020) recently released two spiking datasets using LAUSCHER:

- The Spiking Heidelberg Digits (SHD) dataset contains spoken digits from 0 to 9 in both English and German (20 classes). The recordings are from twelve different speakers, two of which are only present in the test set. The train set contains 8,332 examples and the test set 2,088 (there is no validation set).
- The Spiking Speech Commands (SSC) dataset is based on the Google Speech Commands v0.2 dataset and contains 35 classes from a larger number of speakers. The number of examples in the train, validation and test splits are 75,466, 9,981, and 20,382, respectively.

In both datasets, the original waveforms have been converted to spike trains over 700 input channels. These spiking datasets form an adapted benchmark and allow the investigation of SNNs as well as the comparison of different techniques.

The current state-of-the-art on the SHD and SSC datasets is summarized in Tables 2, 3. The SNN methods are presented in the upper section of the tables, and in the lower sections, the non-spiking CNN and LSTM serve as a point of comparison with the ANN performance.

**Table 2:** State-of-the-art on the SHD dataset.

**Method**	**Test acc. (%)**
Attention (Yao et al., 2021)	**91.1**
Recurrent + adaptation (Yin et al., 2021)	90.4
Recurrent + adaptation (Yin et al., 2020)	84.4
Recurrent + data augm. (Cramer et al., 2020)	83.2
Recurrent + heter. time const. (Perez-Nieves et al., 2021)	82.7
Recurrent (Cramer et al., 2020)	71.4
Non-recurrent (Cramer et al., 2020)	47.5
CNN (Cramer et al., 2020)	**92.4**
LSTM (Cramer et al., 2020)	89

Bold values indicate the best performing network of its category (ANN or SNN).

**Table 3:** State-of-the-art on the SSC dataset.

**Method**	**Test acc. (%)**
Recurrent + adaptation (Yin et al., 2021)	**74.2**
Recurrent + heter. time const. (Perez-Nieves et al., 2021)	57.3
Recurrent (Cramer et al., 2020)	50.9
Non-recurrent (Cramer et al., 2020)	41.0
CNN (Cramer et al., 2020)	**77.7**
LSTM (Cramer et al., 2020)	73

Bold values indicate the best performing network of its category (ANN or SNN).

#### 2.6.3. Non-spiking datasets

**Figure 3:**
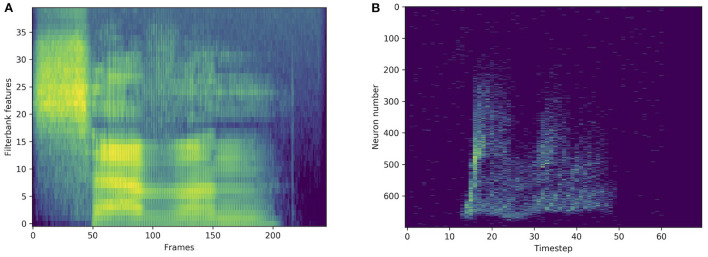
Standard representation *via* filterbank features **(A)** and spike train representation *via* LAUSCHER **(B)** of the same spoken digit (seven in English) from the SHD dataset.

The original, non-spiking versions of the SHD and SSC datasets are available and will also be considered in this work. For the Heidelberg Digits (HD) and Google Speech Commands (SC) datasets, acoustic features are extracted from the waveforms and fed into neural networks. An input example is illustrated in Figure 3, where the filterbank and spiking approaches are compared. The second version of the original Speech Commands (SC) dataset introduced by Warden (2018) has the same number of examples as its spiking version (SSC), but different training, validation, and testing splits of 84,843, 9,981, and 11,005 examples, respectively. The SSC has a 70/10/20% partition instead of 80/10/10% for the SC. This makes a direct comparison impossible between the accuracies on the two tasks, as the SC has considerably more training data. For the HD and SHD datasets however, the splits are the same. We were not able to find state-of-the-art results on the non-spiking HD dataset, however, for the SC dataset, the state-of-the-art is presented in Table 4. Note that certain approaches, such as that of Pellegrini et al. (2021), use the first version of the SC dataset. Others, like Zhang et al. (2017) or Rybakov et al. (2020) do use the second version of the dataset, but only 12 labels instead of 35, by using an “unknown” category that includes some of the remaining words. This explains the absence of some of the literature inside the state-of-the-art table, as their results unfortunately cannot directly be compared with ours. In all state-of-the-art tables, the best test accuracies by SNNs and ANNs are written in bold.

**Table 4:** State-of-the-art on the SC dataset (version 2 with 35 labels).

**Method**	**Test acc. (%)**
Recurrent + adaptation (Salaj et al., 2021)	**91.21**
Recurrent + adaptation (Shaban et al., 2021)	91
Transformers (Gong et al., 2021)	**98.11**
Attention RNN (De Andrade et al., 2018)	93.9

Bold values indicate the best performing network of its category (ANN or SNN).

## 3. Results

We present results for the LIF and adLIF neuron models defined in Sections 2.1.1 and 2.1.2. The more complex, nonlinear Izhikevich and Adex models defined in Section 2.1.3 did not bring improvements over the linear adaptive LIF and are left out of this analysis. We also distinguish between models with and without recurrent connections, in the form of a weight matrix *V* applied to a layer-wise feedback as defined in 2.3, so that SNNs without recurrent connections are considerably lighter in terms of number of trainable parameters. The hidden size of a network corresponds to the number of neurons in each of the hidden layers. Although different hidden layers can have different sizes, we focused on hidden layers of the same size in this study. The number of layers is the number of hidden layers plus one (the readout layer). Networks of increasing size and depth were investigated by varying the hidden size from 128 to 1,024 neurons per layer, and the number of layers from 2 to 5. Overall, three-layered architectures appeared as the best compromise between size and performance and are used in all presented results (with both SNNs and ANNs). Nevertheless, increasing the number of layers showed that the training of the networks was robust to considerable depth. The chosen approach was therefore able to discard scalability limitations of SNNs on these four tasks, which is very encouraging for the compatibility of SNNs with modern deep learning frameworks. In the following sections dedicated to each of the four tasks, the results with SNNs will be presented in the upper region of the tables. We distinguish between the following types of network:

1. tandem: non-recurrent network of non-adaptive IF neurons (no leak) trained with tandem learning rule.
2. LIF: non-recurrent network of non-adaptive LIF neurons trained with a surrogate gradient.
3. adLIF: non-recurrent network of adaptive linear LIF neurons trained with a surrogate gradient.
4. RLIF: recurrent network of non-adaptive LIF neurons trained with a surrogate gradient.
5. RadLIF: recurrent network of adaptive linear LIF neurons trained with a surrogate gradient.

Based on *ad-hoc* experiments, all presented surrogate gradient SNNs use (i) trainable neuron parameters within fixed ranges of values (α for LIF and α, β, *a* and *b* for adLIF neurons as defined in Section 2.3), (ii) a surrogate gradient with the boxcar function as defined in Section 2.4.3, and (iii) a non-spiking readout layer with a cumulative sum over time as defined in Section 2.5. On the other hand, for the ANN baseline, the following types of network will be presented in the lower section of the tables of results:

1. MLP: a simple feed-forward network without recurrence
2. RNN: a standard recurrent network
3. liBRU: a network of light Bayesian recurrent units
4. GRU: a network of gated recurrent units

The liBRU is a probabilistic version of the liGRU with a Softplus activation function instead of a rectified linear unit. We show results with the liBRU instead of the liGRU, as they were slightly better on all four tasks. In terms of number of trainable parameters, on the one hand, tandem, LIF, and adLIF networks are roughly equivalent to MLPs, and on the other hand, RLIF and RadLIF networks are comparable to standard RNNs. By contrast, gated non-spiking networks are considerably larger than all SNNs, since each gate includes weight matrices of its own. With respectively one and two gates, liBRUs and GRUs contain approximately two and three times as many parameters as RNNs of the same size. LSTMs were also tested, but since their performance was observed to be slightly lower than that of liBRUs and GRUs, they are not mentioned in our experiments. Note that although surrogate gradient SNNs have trainable parameters inside their activation function, the implemented ANNs only use a non-trainable homogeneous activation function, as it is the case in current common practice. The implemented SNNs and ANNs therefore differ with respect to the trainability of their respective activation functions. All presented ANNs and SNNs use dropout with *p* = 0.1 as well as batch normalization (Ioffe and Szegedy, 2015). The only hyperparameters in both artificial and spiking networks are the dropout rate, the learning rate, and the patience and decay factor of its scheduler. Given that we were not initially aiming for state-of-the-art performance, a simple *ad-hoc* search was carried out to tune them. Convergence curves can be found in Supplementary material (Supplementary Figures 1–4).

### 3.1. Spoken digit recognition on SHD

Owing to its small size, the SHD data set allows a thorough investigation of the best choice of architecture. On this specific task, we show for the first time that SNNs can surpass ANNs. Our results are illustrated in Table 5. First notice that our best SNN results are better than the attention based SNN state of the art of 91.1% by Yao et al. (2021) (see Table 2). More importantly, our approach also improves upon the best reported ANN-performance of 92.4% by Cramer et al. (2020), which used a convolutional neural network (CNN). Our own attempts with recurrent ANNs only reached 90.40% with GRUs. Even using non-recurrent connections and a relatively small network (3 × 128), we obtained an accuracy of 93.06% with adaptive spiking neurons. This shows a remarkable ability of SNNs to compete with much larger standard networks. With recurrence and a higher number of neurons, our best performing SNN even reached a test accuracy of 94.62%, which is extremely promising for the future of spiking networks with surrogate gradients.

**Table 5:** Results on the SHD dataset.

**Network**	**Recurrent**	**Number**	**Hidden**	**Test**
**type**	**connections**	**of layers**	**size**	**accuracy (%)**
Tandem	No	3	128	62.64
			1024	68.01
LIF	No	3	128	87.04
			1024	89.29
adLIF	No	3	128	93.06
			1024	93.57
RLIF	Yes	3	128	89.75
			1024	92.51
RadLIF	Yes	3	128	92.88
			1024	**94.62**
MLP	No	3	128	61.63
RNN	Yes	3	128	73.48
liBRU	Yes	3	128	89.61
GRU	Yes	3	128	**90.40**
SNN SOTA (Yao et al., 2021)				91.1
ANN SOTA (Cramer et al., 2020)				92.4

The number of successes or positives in an experiment with binary outcomes can be modeled as a binomial distribution. The posterior of the binomial parameter is beta distributed for trivial priors. Here, the equal tailed 95% credible intervals for a flat prior are between ±2.1 and ±0.9% for test set accuracies between 61.63 and 94.62%. Note that larger ANNs were also tested but only obtained slight improvements and remained under the performance of SNNs of the same size. Bold values indicate the best performing network of its category (ANN or SNN).

We also tested the tandem approach of Wu et al. (2021) presented in 2.4.1, which is an alternative to surrogate gradients. This method so far does not allow recurrent connections. Even if the results are slightly higher (62.64%) than those with a MLP (61.63%), they are significantly lower than what we get with the surrogate gradient approach for a network of the same size (87.04 and 93.06% for LIF and adLIF neurons, respectively). This can be seen as evidence of the importance of using the precise spike timings inside the training mechanism.

### 3.2. Spoken digit recognition on HD

In order to compare with standard methods for speech recognition, some experiments were made on the original, non-spiking Heidelberg digits (HD) dataset. Filterbank features were extracted from the waveforms, and directly fed into various networks. As illustrated in Figure 3, compared to a spiking input generated with LAUSCHER, which is a 700 neurons × 100 timesteps sparse binary tensor, here a non-spiking input typically takes the form of a 40 features × 250 frames real-valued tensor. Even though the first hidden layer receives real-valued sequences instead of spike trains, spiking networks remain compatible with this approach. They even outperform their non-spiking equivalents, as presented in Table 6, where the LIF and RLIF networks surpass the MLP and RNN, respectively. The light Bayesian recurrent unit (liBRU) was also tested and gave the best overall performance, although it requires roughly twice as many trainable parameters as a RNN or RLIF network. The accuracies reached with this filterbank approach are considerably higher than the ones on the spiking dataset. Most investigated models were able to reach a test accuracy close to 100%, which is why we only show a few relevant results. This seems to indicate that some information is lost when performing the conversion from waveform to spikes with LAUSCHER, compared to the extraction of acoustic features. Here the conversion from filterbank features to spike trains happens in a trainable fashion inside the neuronal dynamics of the first hidden layer. Moreover, the initial (non-trainable) transformation of the audio waveforms into filterbank features is fast enough to be performed during training, so that our approach with the non-spiking data sets does not require any preliminary processing of the audio, and could be suitable for low-powered hardware implementations.

**Table 6:** Results on the HD dataset.

**Network**	**Recurrent**	**Number**	**Hidden**	**Test**
**type**	**connections**	**of layers**	**size**	**accuracy (%)**
LIF	No	3	128	98.40
RLIF	Yes	3	128	**99.35**
MLP	No	3	128	96.99
RNN	Yes	3	128	99.13
liBRU	Yes	3	128	**99.96**
GRU	Yes	3	128	99.91

Here the equal tailed 95% credible intervals are between ±0.5 and ±0.1% for test set accuracies between 96.99 and 99.96%. Bold values indicate the best performing network of its category (ANN or SNN).

### 3.3. Speech command recognition on SSC

The SSC dataset is roughly ten times bigger than the SHD and has 35 labels instead of 20. It already represents a more complicated classification task to solve for a neural network. Our results are presented in Table 7. Here, we managed to close the gap between the SNN and ANN performances by reaching a test accuracy of 77.4% with an SNN. Even though this already represents considerable improvements upon the best previously reported SNN result of 74.2% by Yin et al. (2021), our results remain slightly lower than the (non-spiking) CNN performance of 77.7% reported by Cramer et al. (2020), and also lower than our best ANN-performance of 79.05% with GRUs. Nevertheless, in terms of number of trainable parameters, if we compare SNNs to ANNs of the same size, the LIF and adLIF networks score substantially better (66.67 and 71.66%) than the MLP (only 29.27%), and the RLIF and RadLIF outperform (73.87 and 73.25%) the RNN (70.01%).

**Table 7:** Results on the SSC dataset.

**Network**	**Recurrent**	**Number**	**Hidden**	**Test**
**type**	**connections**	**of layers**	**size**	**accuracy (%)**
LIF	No	3	128	66.67
			512	68.14
adLIF	No	3	128	71.66
			512	73.58
RLIF	Yes	3	128	73.87
			512	75.91
RadLIF	Yes	3	128	73.25
			512	76.21
			1024	**77.40**
MLP	No	3	128	29.27
RNN	Yes	3	128	70.01
liBRU	Yes	3	512	78.70
GRU	Yes	3	512	**79.05**
SNN SOTA (Yin et al., 2021)				74.2
ANN SOTA (Cramer et al., 2020)				77.7

Here the equal tailed 95% credible intervals on the accuracies are all about ±0.6% due to the large size of the test set. Bold values indicate the best performing network of its category (ANN or SNN).

### 3.4. Speech command recognition on SC

Our results on the non-spiking SC dataset are presented in Table 8. We find that our approach is able to reach even better accuracies than the current SNN state-of-the-art of 91.21% by Salaj et al. (2021), which also uses recurrent SNNs, but with a different model of adaptation. We also find that the chosen SNN approach surpasses the performance of almost all implemented ANNs. With a similar number of trainable parameters, the non-recurrent LIF and adLIF networks give much better results (82.12 and 90.46%) than the MLP which only scores 48.80% on this task. Similarly, the recurrent RLIF and RadLIF networks achieve accuracies of 90.71 and 92.48%, respectively, compared to 90.61% for a non-spiking equivalent RNN. For larger units, we even observe that a non-recurrent, adaptive SNN (adLIF) is able to outperform a conventional RNN with 93.12% against 92.09%. This illustrates the advantage of physiologically plausible spiking neuron models as the former is significantly lighter than the latter in terms of trainable parameters. By adding recurrence and a larger number of hidden units, we find that our best performing SNN (94.51%) even surpasses the Attention RNN approach of De Andrade et al. (2018) (93.9%), which remained as the ANN state-of-the-art on this task for a long time. With roughly twice as many trainable parameters, the liBRU is the only ANN in our baseline that is able to modestly exceed the RadLIF SNN performance. More generally, the SNN approach appears able to compete with state-of-the-art gated recurrent networks, whilst retaining a definitive advantage of energy efficiency; this is extremely encouraging for further work in this direction.

**Table 8:** Results on the SC dataset.

**Network**	**Recurrent**	**Number**	**Hidden**	**Test**
**type**	**connections**	**of layers**	**size**	**accuracy (%)**
LIF	No	3	128	82.12
			512	83.03
adLIF	No	3	128	90.46
			512	93.12
RLIF	Yes	3	128	90.71
			512	93.58
RadLIF	Yes	3	128	92.48
			512	**94.51**
MLP	No	3	128	48.80
			512	53.16
RNN	Yes	3	128	90.61
			512	92.09
liBRU	Yes	3	128	94.55
			512	**95.06**
GRU	Yes	3	128	93.65
			512	94.32
SNN SOTA (Salaj et al., 2021)				91.21
ANN SOTA (Gong et al., 2021)				98.11

Here the equal tailed 95% credible intervals are between ±0.9 and ±0.4% for test set accuracies between 48.80 and 95.06%. Bold values indicate the best performing network of its category (ANN or SNN).

### 3.5. Reducing the network size

So far in this study, we have only considered fully connected layers as it was the most general case and allowed a direct comparison with standard ANNs. The number of trainable parameters can be an important limitation in energy efficient implementations of neural networks. The contributions of the different trainable components used in our spiking architectures are listed here below for a layer *l* with *N*^*l*^ hidden units.

- Feedforward weights: *N*^*l*−1^·*N*^*l*^
- Recurrent weights: *N*^*l*^·*N*^*l*^
- Biases: *N*^*l*^
- LIF neuron parameters (α): *N*^*l*^
- adLIF neuron parameters (α, β, *a*, *b*): 4·*N*^*l*^

We see that the main contribution to the total number of trainable parameters comes from the weights. Imposing a lower connectivity can therefore greatly reduce the network size. This is especially effective in the first layer on the spiking datasets due to the very high number of input neurons (*N*^0^ = 700). Further experiments with a sparser connectivity in the first layer have been carried out on the SHD dataset, and are presented in Table 9. We observe that gradually reducing the connectivity in the first layer only hinders the accuracy by about 1.5%, even when randomly removing up to 99% of the connections. Other experiments presented in Table 10 were made with large RadLIF networks. Here a portion of both feedforward and recurrent connections was randomly removed in all hidden layers. We see that the sparser networks are still able to achieve state-of-the-art accuracies, and that even reducing the number of weights by a factor of 20 only decreases the accuracy by about 1.4%.

**Table 9:** Results on the SHD dataset for a non-recurrent adLIF network of size 3 × 128, with a sparser connectivity in the first layer.

**Sparsity**	**Number of**	**Test**
**proportion (%)**	**trainable parameters**	**accuracy (%)**
0	109,864	93.06
10	100,904	92.83
50	65,064	92.14
90	29,224	92.33
95	24,744	92.14
99	21,160	91.59

Here a sparsity proportion *p* corresponds to randomly removing *p%* of the connecting weights in the first layer.

**Table 10:** Results on the SHD dataset for a recurrent RadLIF network of size 3 × 1,024, with a sparser connectivity in all hidden layers.

**Sparsity**	**Number of**	**Test**
**proportion (%)**	**trainable parameters**	**accuracy (%)**
0	3,893,288	94.62
10	3,507,035	93.80
50	1,962,024	93.57
90	417,013	92.51
95	223,886	93.20
99	69,385	90.95

Here, a sparsity proportion *p* corresponds to randomly removing *p%* of the connecting weights (both feedforward and recurrent) in all hidden layers.

Another way of reducing the size of the network is through the parameters of the spiking neuron model. We distinguished four cases in *ad-hoc* experiments: (i) fixed and homogeneous, i.e., the same fixed value for all neurons in the layer, (ii) fixed and heterogeneous, i.e., distributed but fixed values for all neurons in a layer, (iii) trainable and homogeneous, i.e., having a single trainable parameter shared by all neurons in the same layer, and finally (iv) trainable and heterogeneous, i.e., each neuron has its own trainable parameters. We found that the fourth case gave significantly better results and was therefore chosen for the whole paper. As demonstrated by Perez-Nieves et al. (2021), the heterogeneous nature of a spiking layer allows each neuron to develop its own “activation function,” defined by the values of its parameters. This appears as one core advantage over conventional ANN models in which the same activation function is shared by all units. We believe that this neural heterogeneity contributes to the superior representational capacities of spiking neurons when applied to auditory sequences.

## 4. Discussion

### 4.1. Toward physiological plausibility

Different neuron models were investigated in our study. In order of increasing complexity and physiological plausibility: the leaky integrate-and-fire (LIF), adaptive linear LIF (adLIF), adaptive quadratic LIF (Izhikevich), and adaptive exponential LIF (AdEx), described in Section 2.1. As presented in the results Section 3, adding adaptation with the adLIF model consistently improved the performance compared to the LIF. Nevertheless, the more physiological nonlinear AdEx and Izhikevich models (that were left out of the presented results) did not achieve better performances than the simpler linear adLIF model. This could be an effect of the weaker compatibility of these inherently more complex models with gradient descent. The spiking datasets we use may also not be biologically plausible enough to take advantage of those models.

In this work, adaptation is described by the discrete time Equations (7) to (9), that are not based on a moving threshold, but on subthreshold coupling and spike-triggered currents. These directly stem from the continuous time formulation of Equations (3) and (4), as defined in Gerstner and Kistler (2002), which represent a linear version of the quadratic or Izhikevich neuron model (Izhikevich, 2007). Moreover, Mensi et al. (2012) have shown that depending on the type of cortical neurons, spike-frequency adaptation was dominantly mediated by spike-triggered currents or moving threshold. Although both produce spike-frequency adaptation, they inherently represent different mechanisms. A fair amount of the reported approaches have used adaptive neurons on the studied datasets (Yin et al., 2020, 2021; Salaj et al., 2021; Shaban et al., 2021). However, they all employ a moving threshold formulation of adaptation, that is similar to that of Bellec et al. (2018), in which the dynamical threshold is specific to each neuron and increases by a fixed amount after firing, before decaying back to some rest value. Note that Shaban et al. (2021) actually use a more complex version of the adaptive threshold that includes a second time constant. Nevertheless, these are all based on a moving threshold adaptation model. In order to test whether the improvements came from our specific implementation of adaptation, several experiments were made using the moving threshold formulation. A comparison between the two approaches is presented in Table 11. We observe that our formulation of adaptation significantly outperforms the moving threshold alternative, especially on the SHD dataset.

**Table 11:** Comparison between our adaptation scheme and an alternative moving threshold on the SHD and SC datasets.

**Dataset**	**Network**	**Moving threshold (%)**	**Spike-triggered currents**
SHD	RadLIF 3 × 128	88.79	**92.87**
SHD	RadLIF 3 × 512	90.21	**93.75**
SHD	RadLIF 3 × 1,024	89.25	**94.62**
SC	RadLIF 3 × 128	90.95	**92.48**
SC	RadLIF 3 × 512	93.61	**94.51**

Bold values indicate the best performing type of adaptation.

Coming back to Table 8, on the SC dataset, adding adaptation had the same impact as adding recurrent connections, even though the former requires remarkably less trainable parameters than the latter. On the SHD dataset, the effect of adding adaptation is even more pronounced as the considerably lighter adLIF networks scored better than the RLIFs (see Table 5). This shows the importance of the neuron model and, more generally, of a physiologically plausible approach. As pointed out by Perez-Nieves et al. (2021), the heterogeneity of the spiking neurons is a metabolically and computationally efficient strategy. In ANNs, as defined in Equation (11), all neurons have the same activation function. The resulting homogeneity in the behavior of standard artificial neurons implies that the only source of heterogeneity lies in the synaptic connections, that can be different for each neuron. However, adding neurons to the network increases the computational cost by an order of $O\left(N^{2}\right)$ for fully-connected layers. With spiking neurons, the more complex neuronal dynamics allow heterogeneous behaviors among neurons by depending on trainable parameters that only scale with O(N), hence the more efficient computational strategy.

In terms of the learning rule, the compatibility with deep learning methods was favored over the biological plausibility of the approach. Nevertheless, the surrogate gradient technique can actually be a good candidate toward more physiologically plausible learning algorithms. Kaiser et al. (2020) have recently defined a deep continuous local learning rule (DECOLLE) using random readouts at each layer. Their method still uses surrogate gradients to allow SGD, but is closer to a form of bio-inspired plasticity. This seems to indicate that the evaluated compatibility of training SNNs within ANN frameworks could lead to further improvements of the training methods, and allow more physiologically plausible learning rules by retaining the advantages of well-developed ANN techniques.

Finally, compared to the SNN-ANN tandem method of Wu et al. (2021), the chosen surrogate gradient approach does not ignore the spike timings during the backward pass. In addition to being more flexible to easily include recurrence and different neuron models, the latter gave considerably better results than the former, which suggests that the precise timing of the spikes is of importance in processing temporal information.

### 4.2. Toward energy-efficient hardware

In other papers that used the spiking datasets (see Tables 2, 3), non-recurrent SNNs were always reported to perform substantially less well-compared to their recurrent counterparts. In this study, we managed to raise the performance of lighter, non-recurrent SNNs. Our results with non-recurrent adLIF models on the SHD and SC data sets were even able to surpass those of the best previously reported recurrent SNNs on the same tasks. This allows competitive networks with much fewer trainable parameters, and could lead to hardware implementations that require less space, power, and memory.

The average firing rate $\bar{\nu }$ of the implemented spiking networks (over all neurons and all time steps) was observed to consistently converge around $\bar{\nu }\approx 0.1$, which corresponds to 10 Hz. To compare the energy consumption of SNNs with ANNs, similarly to Panda et al. (2020), one can count the number of accumulate (AC) and multiply-and-accumulate (MAC) operations that are required at each time step. Here, we consider ANNs that process sequential inputs and focus on the case with recurrent connections, i.e., RNNs. In contrast to Equation (10) where the matrix multiplications involve non-zero real numbers and results in *N*^*l*^(*N*^*l*−1^+*N*^*l*^+1) MACs for RNNs, Equation (12) only requires $\bar{\nu }N^{l}\left(N^{l-1}+N^{l}+1\right)$ ACs for SNNs. The first energy gain therefore comes from the sparsity of the spike trains in Equation (12), which gets rid of $1-\bar{\nu }\approx 90\%$ of the required operations as most neurons are not activated. Even if the internal neuronal dynamics of SNNs described by Equations (7−9) require additional operations compared to the ANN activation of Equation (11), these only scale with the number of hidden units *N*^*l*^ in the current layer *l*, whereas the benefits of sparsity scale with (*N*^*l*^)^2^.

Moreover, SNNs replace the MACs by ACs in the dot-product computations as a consequence of the binary nature of spike trains, which constitutes the second gain of energy. As presented by Han et al. (2015) with a 45 nm CMOS process, a single 32-bit integer AC operation only requires 0.1 pJ compared to 3.2 pJ for a MAC. This reduces the energy consumption by another factor of 32. Even if they usually lead to slightly worse accuracies, regularizers can additionally be used to obtain even sparser spike trains and reduce the value of $\bar{\nu }$. By combining the advantages of the sparse and binary nature of the information, a recurrent SNN without regularizers already requires roughly 320 times less energy than a non-spiking RNN of the same size.

On the ANN side, this energy gap can nevertheless be reduced. Low footprint keyword spotting techniques involve network quantization (Zhang et al., 2017), a max-pooling based loss function (Sun et al., 2016), cascaded executions (Sun et al., 2015; Giraldo et al., 2019), compute-in-memory architectures (Schaefer et al., 2021), and various specialized hardware (Conti et al., 2018; Giraldo et al., 2020; Kadetotad et al., 2020) designed to reduce the energy consumption of RNNs. Very recently, Jeffares et al. (2022) have notably taken inspiration from SNNs to define a threshold based rank coding approach with considerable speed and efficiency benefits. Even though the efficiency superiority of SNNs can be nuanced by taking into consideration the above ANN methods, they inevitably play a central role in developing lower powered neuromorphic hardware.

The main motivation for using ANNs is their task performance, which is typically superior to that of SNNs, especially when using sophisticated architectures such as gates or attention. In this work however, we have seen that SNNs consistently outperformed non-gated RNNs of the same size. This apparent superiority can be explained by the heterogeneous and trainable activation of spiking neurons, which represents an advantage over the implemented ANNs, that similarly to common practice, use homogeneous activation functions. The higher representational capabilities of using heterogeneous neural activations in SNNs is well-illustrated by the fact that on all four tasks, even the much lighter non-recurrent adaptive SNNs managed to surpass the performance of standard RNNs. Only gated RNNs were able to compete with SNNs and surpass them in most cases. However, layers of liBRUs and GRUs require two and three times more operations respectively compared to standard RNN layers, thus expanding the energy gap even more drastically. The sparse event-driven processing of the information in SNNs, combined with their assessed capabilities therefore make them extremely attractive for reaching lower powered hardware implementations dedicated to real-world applications.

## 5. Conclusion

In the introduction we set out three goals for the work. In concluding, by carefully selecting appropriate techniques, we have established an SNN method that, on top of being compatible with standard deep learning frameworks, is capable of competing with ANNs on the same speech processing tasks, whilst conserving the advantage of energy efficiency. This represents the main contribution of this paper, which in fact fulfills the first goal. The chosen surrogate gradient approach allows SNNs to be trained with gradient descent like conventional ANNs. The resulting compatibility with modern ANN frameworks combined with the observed scalability of our spiking networks to relatively deep architectures point toward further applications of this method to more advanced tasks. In terms of energy consumption, the implemented SNNs are drastically more efficient compared to standard ANNs of the same size, showing promising pathways for low-powered hardware implementations of neural networks.

At the same time, we have also achieved the second goal of assessing the more general capability of SNNs in comparison to conventional ANNs. We have shown that the particular combination of adaptive spiking neurons, surrogate gradients and automatic differentiation can actually compete with strong ANN baselines on speech recognition tasks. Our implementation of adaptation in the neuron model was able to replace recurrent connections at a considerably lower cost in terms of number of trainable parameters. Such lighter non-recurrent SNNs were even capable of competing with much larger, standard gated recurrent units. Whilst the neurons inside such conventional ANNs all share the same activation function, firing behaviors among spiking neurons can become heterogeneous by making the neuron parameters trainable, which appears to allow more complex representations of the temporal information with fewer neurons inside the network. The implemented SNNs were indeed consistently superior to *equivalent* non-spiking architectures, which further corroborates the hypothesis of greater representational capabilities. This also points toward further investigations of heterogeneous activation functions inside ANNs, as they are not commonly used in current practice.

The success of this physiologically plausible approach to modeling neural networks indicates that our more general third and last goal is still valid. The experiments do not attempt to say anything about biological function. However, they show that a representational capability, that is available to biological entities, is capable of solving the same problems as (artificial) networks that are known to be capable of exceeding human performance on many tasks. This provides a strong hypothesis for future understanding of the biological mechanisms of the brain.

## Software

In order to further encourage the development of spiking neural networks, we make our code available open source at https://github.com/idiap/sparch.

## Data availability statement

The data presented in the study are publicly available. The SHD, SSC and HD datasets can be found at https://compneuro.net/datasets/ and the SC dataset at https://www.tensorflow.org/datasets/catalog/speech_commands.

## Author contributions

AB performed the experiments, did much of the literature search, and wrote the bulk of the manuscript. PG secured funding, supervised the experimental work, and assisted with literature and writing. All authors contributed to the article and approved the submitted version.

## Funding

This project received funding under NAST: Neural Architectures for Speech Technology, Swiss National Science Foundation grant 185010 (https://data.snf.ch/grants/grant/185010).

## Conflict of interest

The authors declare that the research was conducted in the absence of any commercial or financial relationships that could be construed as a potential conflict of interest.

## Publisher's note

All claims expressed in this article are solely those of the authors and do not necessarily represent those of their affiliated organizations, or those of the publisher, the editors and the reviewers. Any product that may be evaluated in this article, or claim that may be made by its manufacturer, is not guaranteed or endorsed by the publisher.

## Supplementary Material

Supplementary Figure 1 Convergence plot of the RadLIF network on the SC dataset.

Supplementary Figure 2 Convergence plot of the GRU network on the SC dataset.

Supplementary Figure 3 Convergence plot of the adLIF network on the SHD dataset.

Supplementary Figure 4 Convergence plot of the GRU network on the SHD dataset.
