# Supplementary figures and images for: A surrogate gradient spiking baseline for speech command recognition
Source: Front Neurosci. 2022 Aug 22;16:865897. doi: 10.3389/fnins.2022.865897 (PMC9479696; doi:10.3389/fnins.2022.865897)

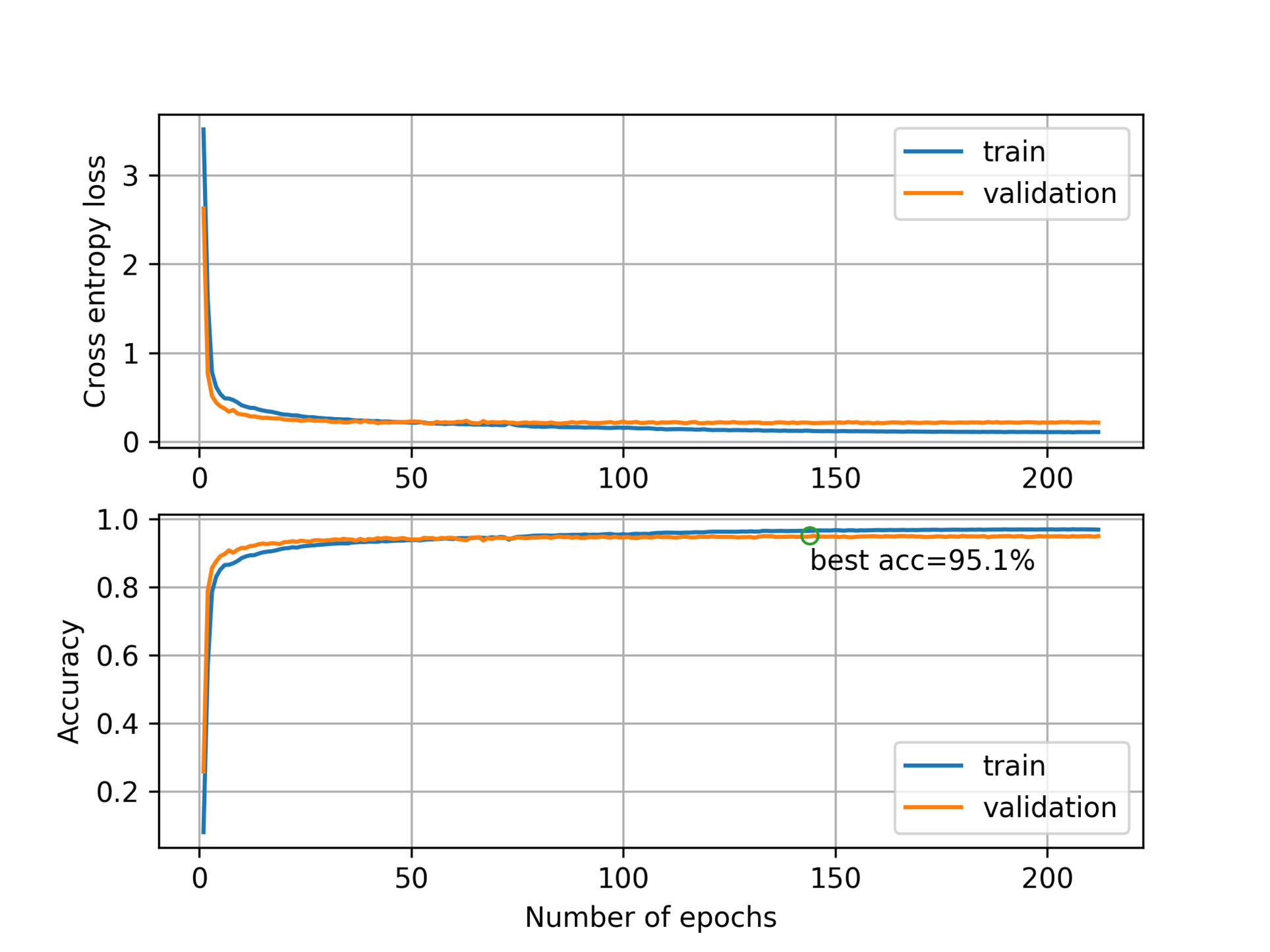

Supplement: Supplementary Figure 1 — Convergence plot of the RadLIF network on the SC dataset. [file Image_1.PNG]

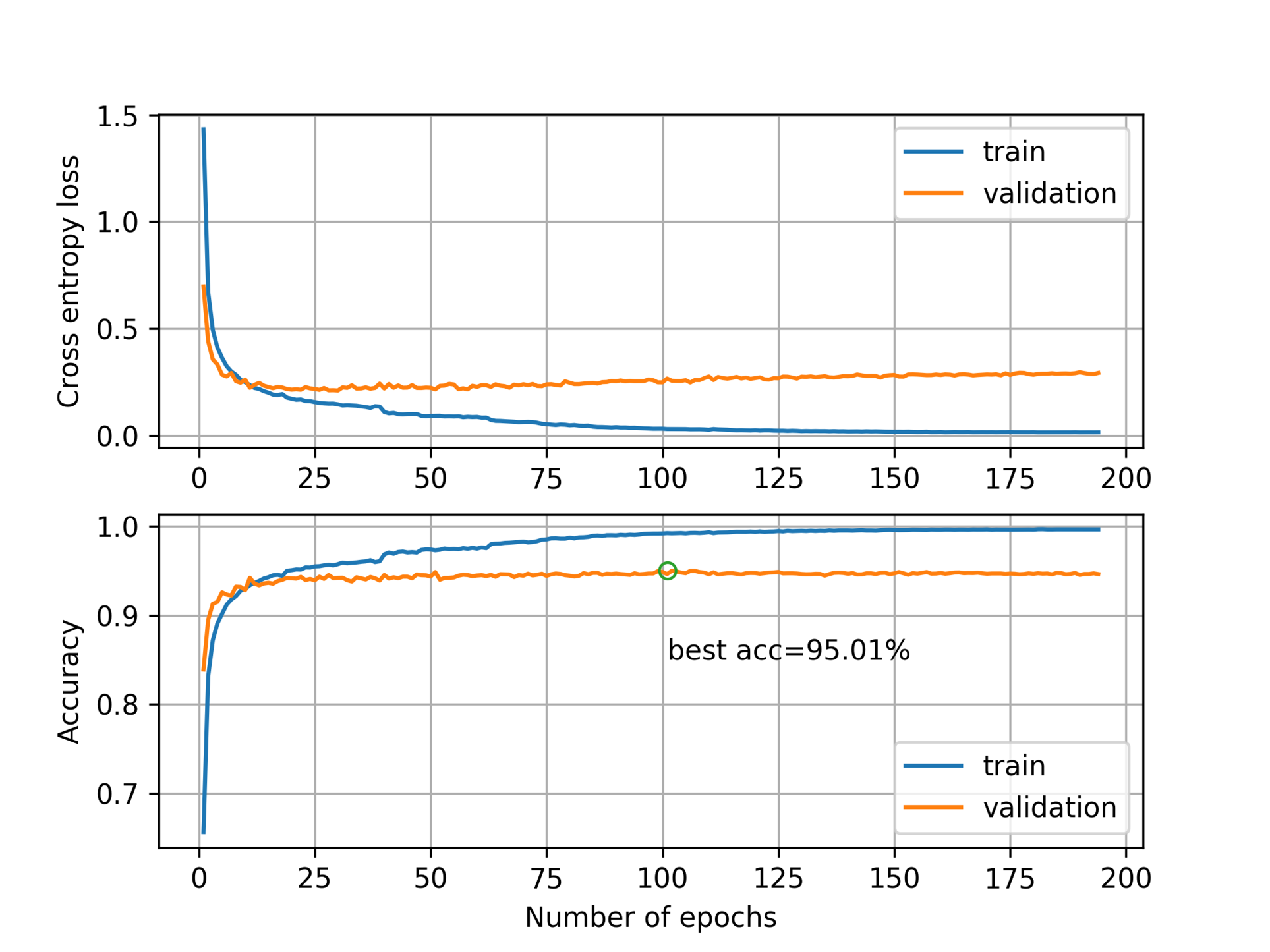

Supplement: Supplementary Figure 2 — Convergence plot of the GRU network on the SC dataset. [file Image_2.PNG]

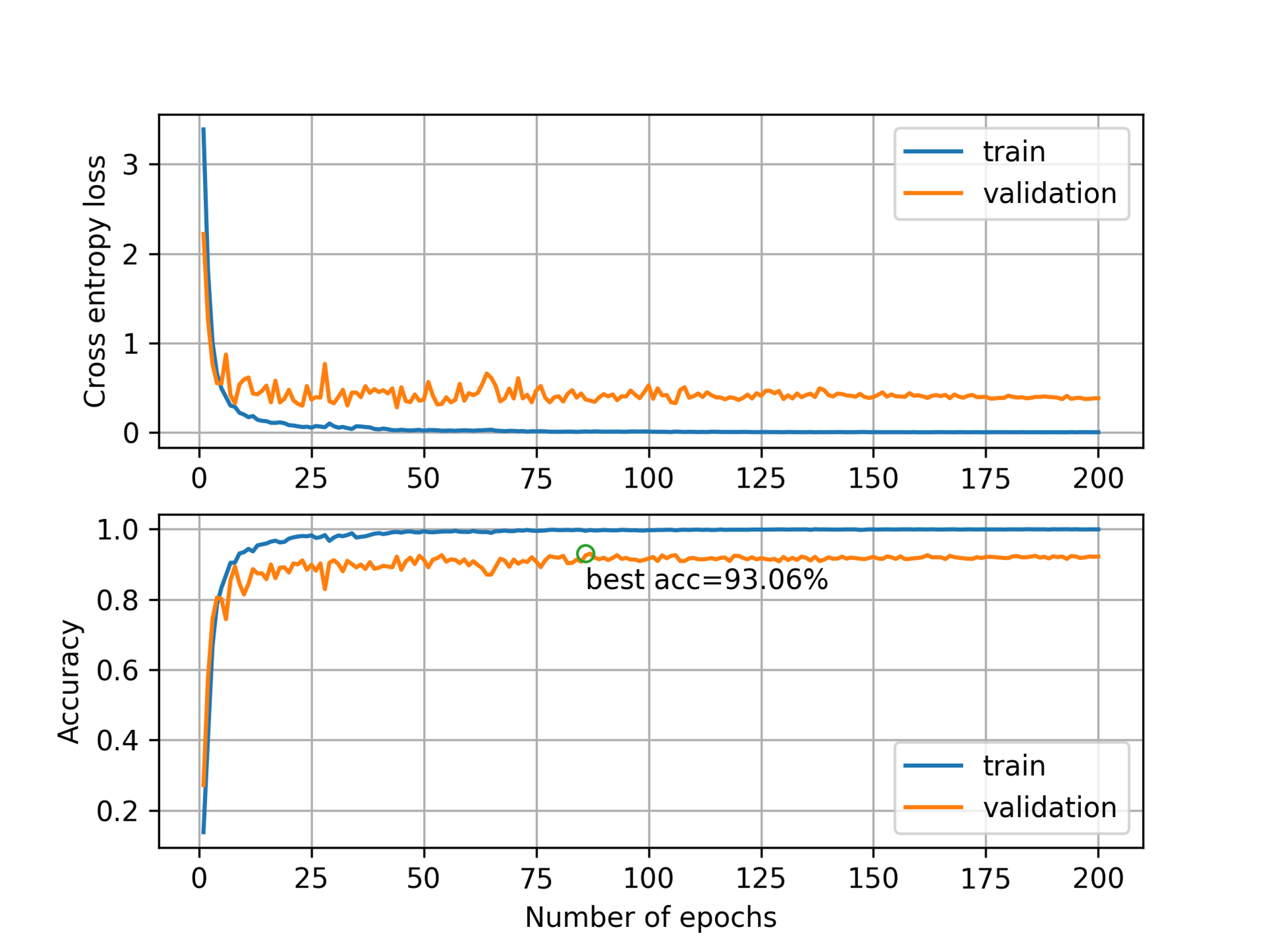

Supplement: Supplementary Figure 3 — Convergence plot of the adLIF network on the SHD dataset. [file Image_3.PNG]

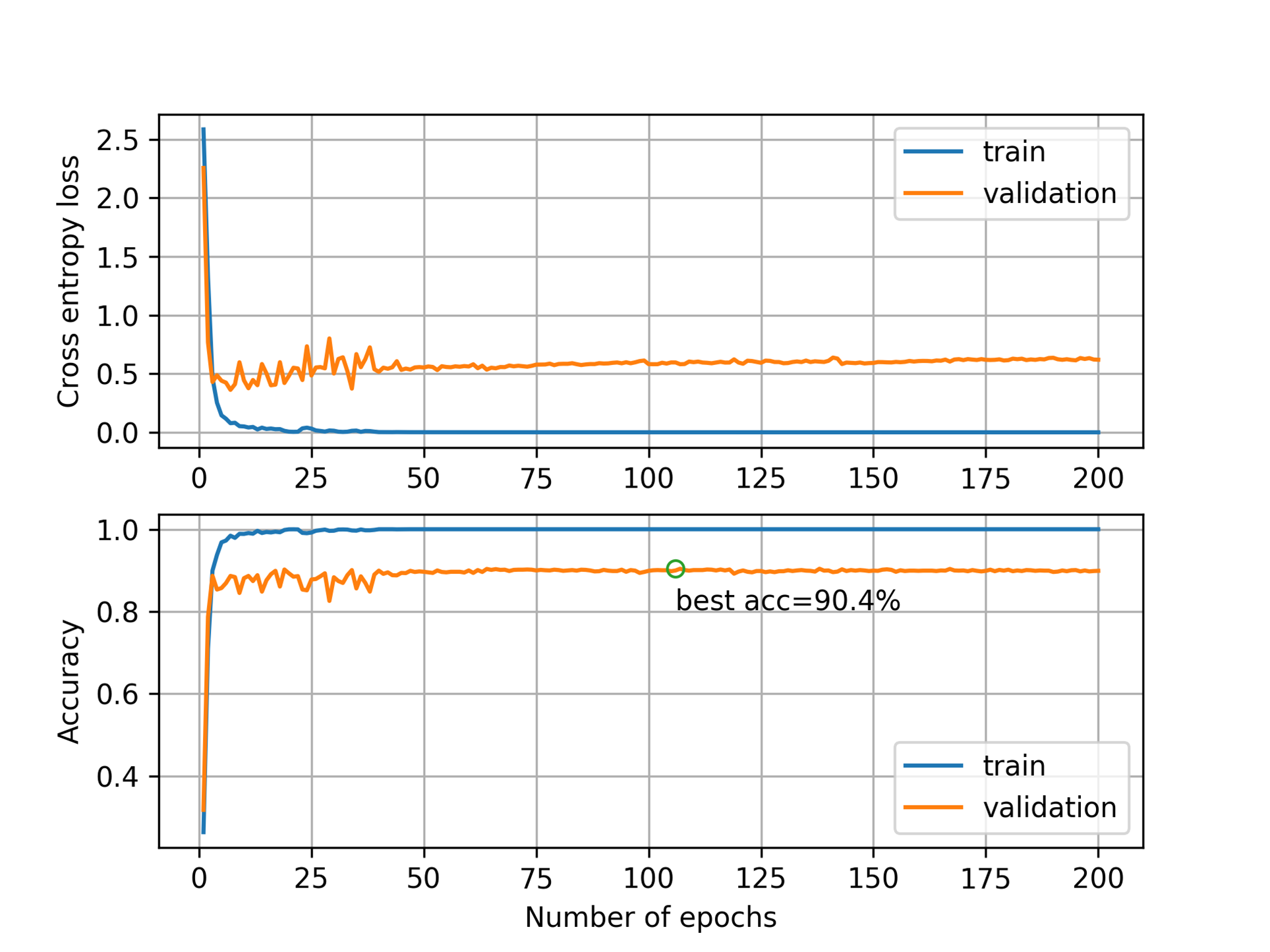

Supplement: Supplementary Figure 4 — Convergence plot of the GRU network on the SHD dataset. [file Image_4.PNG]
